# DNA-mediated cooperativity facilitates the co-selection of cryptic enhancer sequences by SOX2 and PAX6 transcription factors

**DOI:** 10.1093/nar/gku1390

**Published:** 2015-01-10

**Authors:** Kamesh Narasimhan, Shubhadra Pillay, Yong-Heng Huang, Sriram Jayabal, Barath Udayasuryan, Veeramohan Veerapandian, Prasanna Kolatkar, Vlad Cojocaru, Konstantin Pervushin, Ralf Jauch

**Affiliations:** 1 Laboratory for Structural Biochemistry, Genome Institute of Singapore, Singapore 138672, Singapore; 2 Donnelly Centre for Cellular and Biomolecular Research, University of Toronto, Toronto, Ontario M5S 3E1, Canada; 3 School of Biological Sciences, Nanyang Technological University, Singapore 637551, Singapore; 4 Genome Regulation Laboratory, Guangzhou Institutes of Biomedicine and Health, Chinese Academy of Sciences,190 Kai Yuan Avenue, Science Park, Guangzhou 510530, China; 5 Integrated Program in Neuroscience, McGill University, Montreal, Quebec H3G 0B1, Canada; 6 University of Chinese Academy of Sciences, No. 19A Yuquanlu, Beijing 100049, China; 7 Qatar Biomedical Research Institute, Qatar Foundation, PO Box 5825, Doha, Qatar; 8 Computational Structural Biology Laboratory, Department of Cell and Developmental Biology, Max Planck Institute for Molecular Biomedicine, Röntgenstrasse 20, Münster 48149, Germany

## Abstract

Sox2 and Pax6 are transcription factors that direct cell fate decision during neurogenesis, yet the mechanism behind how they cooperate on enhancer DNA elements and regulate gene expression is unclear. By systematically interrogating Sox2 and Pax6 interaction on minimal enhancer elements, we found that cooperative DNA recognition relies on combinatorial nucleotide switches and precisely spaced, but cryptic composite DNA motifs. Surprisingly, all tested Sox and Pax paralogs have the capacity to cooperate on such enhancer elements. NMR and molecular modeling reveal very few direct protein–protein interactions between Sox2 and Pax6, suggesting that cooperative binding is mediated by allosteric interactions propagating through DNA structure. Furthermore, we detected and validated several novel sites in the human genome targeted cooperatively by Sox2 and Pax6. Collectively, we demonstrate that Sox–Pax partnerships have the potential to substantially alter DNA target specificities and likely enable the pleiotropic and context-specific action of these cell-lineage specifiers.

## INTRODUCTION

A primary goal of genomics and associated high-throughput technologies is to decipher the *cis*-regulatory code that determines when, where and how sets of genes are expressed. This regulatory code directs biological processes such as embryonic development and differentiation and is the cause for phenotypic variance and evolutionary innovation. Likewise, aberrations in this code can lead to developmental abnormalities and/or disease progression. The location of *cis*-regulatory ‘enhancer’ DNA sequences that govern cellular identities is now known in many cell types and lists of transcription factors (TFs) that bind such enhancers and associated epigenetic signatures are expanding rapidly (1,2). Enhancers are often composed of clustered DNA elements with affinity for a set of TFs. The architecture of an enhancer, that is, the sequence of the DNA elements, their number, relative orientation and spatial arrangement is thought to determine enhancer activity (3,4). However, the process of how TFs in a combinatorial fashion select enhancers to regulate gene expression remains only superficially understood (5,6). TF interactions are dynamic, transient and often depend on cellular and genomic contexts (7). Both, complementary protein interaction surfaces and the sequence of composite DNA motifs mediate TF associations (8). So far, the structural architectures of very few TF–TF interactions have been described at atomic resolution. Some well-studied instances of heterodimeric TF partnerships include PPAR-γ-RXR-α (9), Pax5-Ets (10), AR–FoxA1 (11), Sox2-Oct4 (12,13) as well as Sox17-Oct4 (14–16). In some cases, TF dimerization profoundly alters sequence specificities of the binding partners as demonstrated by the association of the *Drosophila* co-factor extradentrical (Exd) with Hox proteins (17). The detection of such composite DNA-binding sites is computationally challenging, as many conventional motif discovery tools fail to take into account cooperative interactions and allosteric effects that may occur between interacting TFs.

To study how TFs team up to ‘read’ *cis*-regulatory information, we set out to biochemically dissect how members of the *Sry*-related box (Sox) and *pairedbox* (Pax) families of TFs pair off to recognize enhancer sequences with non-canonical ‘cryptic’ TF motif half-sites. All 20 Sox factors encoded in mouse and human genomes are composed of a 79 amino acid L-shaped high-mobility group domain (HMG) that mediates sequence-specific binding to the minor groove of the DNA leading to a pronounced kink (18–20). The first functional target found to be regulated by a Sox TF was the δ-*crystallin* gene within the presumptive lens ectoderm of the chicken embryo (21). A 30-bp core enhancer termed *DC5* located within the third intron drives expression of δ-*crystallin* (22). After the discovery that SoxB1 proteins, in particular Sox2, bind and activate the *DC5* enhancer, it took some time until the identity of a collaborating factor initially termed δEF3 could be uncovered (23). Eventually, δEF3 was found to be Pax6 (24). The chicken *DC5* sequence can also effectively drive reporter gene expression in the *Drosophila* eye and cooperatively recruit homologous fly TFs, suggesting a deep phylogenetic conservation of the Sox2/Pax6 partnership and the regulatory circuit of lens specification (25). Pax6 had been known for some time to be a key regulator of many aspects of development, especially of neurogenesis (26). In addition, Pax6 initiates eye development as remarkably testified when it could be shown that its ectopic expression can induce eye structures in the legs of flies (27). The Pax gene family consists of nine members in mammals, all of which are key developmental TFs (28). Pax proteins are composed of a bipartite 128 amino acid paired (PRD) DNA-binding domain (DBD) consisting of N-terminal PAI and C-terminal RED subdomains binding the major groove of DNA and additionally latched into the minor groove via an extended linker (29,30). Some Pax TFs, including Pax6, contain an additional homeodomain C-terminal of the paired domain (31). This modular domain structure in Pax6 enables the accommodation of diverse sets of binding sites by mechanisms that can include an intricate interplay of the subdomains and alternate utilization of subdomains for the recognition of specific target sites (32–35).

**Figure 1.**
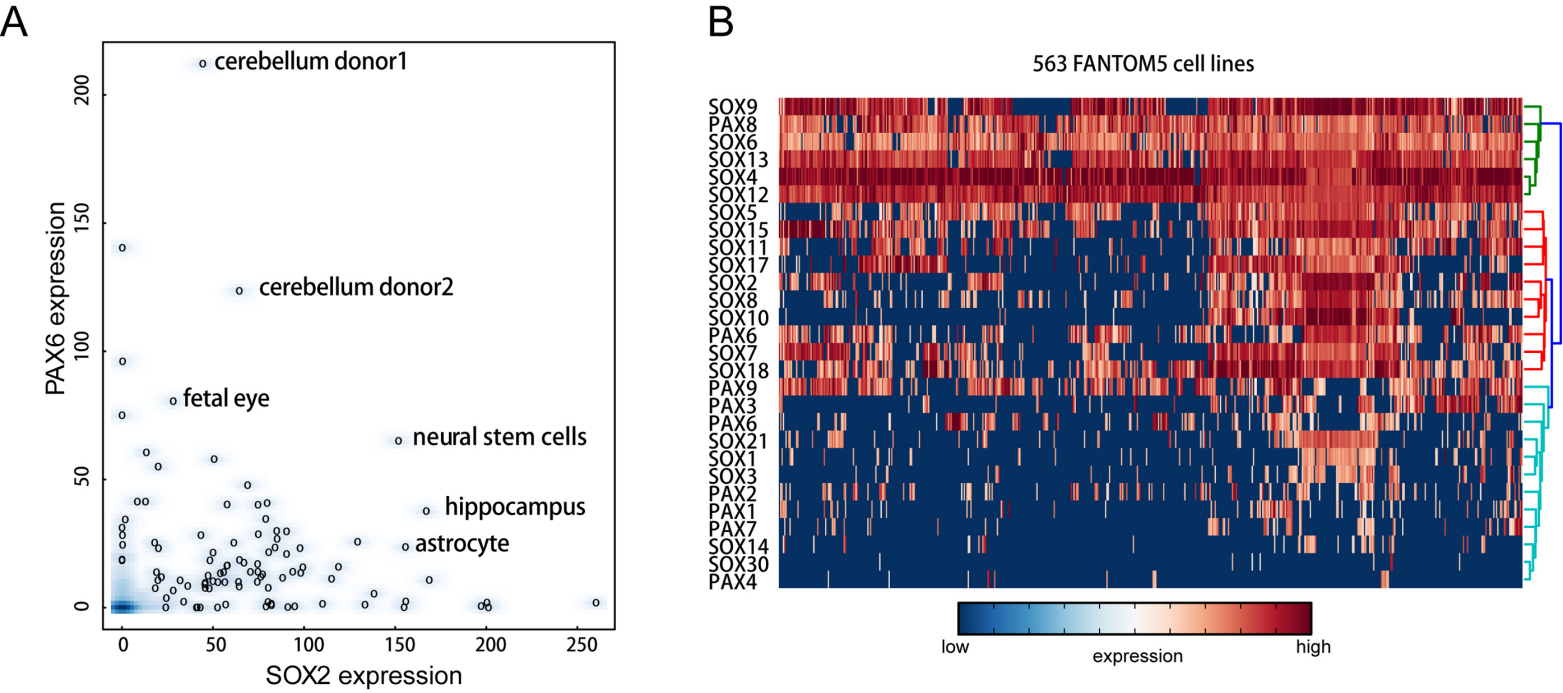
Sox and Pax TFs are co-expressed in many cell types. (**A**) A scatterplot to compare SOX2 and PAX6 expression, highlighting co-expression in several human cell lines. (**B**) A clustered heatmap of 5′ RNA CAGE counts was plotted using glbase with row normalization (44). Red and blue color indicate high and low expression, respectively. Columns are 563 human FANTOM5 cell types. Rows are human SOX and PAX TFs.

In chicken embryos, Pax6 is initially broadly expressed in the head ectoderm and becomes subsequently restricted to the lens placode (24). Inductive signals derived from the optic vesicle induce Sox2/Sox3 proteins in the lens placode cells leading to the activation of δ-*crystallin* expression synergistically with Pax6 (24). A similar cooperation between Sox2 and Pax6 has been implicated in autoregulation of Sox2 by binding to an enhancer element termed *N3*, which is well conserved across vertebrates (36). Both, the *N3* and *DC5* enhancers are highly compact and possess canonical *sox* motifs, but highly degenerate *pax* motifs (36). Yet, this degeneracy is necessary for cooperative binding of Sox2 and Pax6 and for effective activation of target gene expression (24,36). Mutations of *sox* or *pax* half-sites as well as altered motif configurations abolished δ-*crystallin* expression (24). In mouse and humans, both, Sox2 and Pax6, have been demonstrated to be key regulators of neurogenesis (26,37–38). Sox2 and Pax6 are co-expressed in neural stem cells (NSCs), the developing eye and several other neuroectoderm-derived cells, providing ample opportunities for Sox2 and Pax6 to cooperatively bind to target DNA sites in the human genome, reminiscent of the chicken *DC5* and *N3* enhancers (Figure 1A). Additional Sox–Pax partnerships including Sox10 and Pax3 have also been described (39) and the co-expression of Sox and Pax family members is observed in a plenitude of cell types (Figure 1B). We therefore decided to interrogate the biochemical basis for the cooperation of Sox and Pax TFs to (i) uncover the sequence determinants for the cooperative interactions, (ii) understand the biochemical and structural basis for the cooperative formation of ternary complexes, (iii) ask if there is a Sox–Pax partner code that determines target gene selection, (iv) assess whether mutations within Sox2 and Pax6 causative of congenital human eye disorders alter Sox2–Pax6 cooperativity and (v) identify novel Sox/Pax target site in the human genome that supports cooperative interactions.

## MATERIALS AND METHODS

### Cloning, protein production and site-directed mutagenesis

Proteins encoding 79 amino acid HMG domains of mouse Sox2, Sox4, Sox5, Sox15, Sox17 and an extended 109 amino acid Sox2 were used with their amino acid sequences, cloning and purification procedures as published elsewhere (16). The extended 109 amino acid Sox2-HMG construct was chosen for TROSY studies to detect potential interactions in the extended C-terminal tail. This longer Sox2 construct was N^15^ isotope labeled, by growing *Escherichia coli* cells in M9 minimal media containing isotope-labeled ammonium chloride (Cambridge Isotope Laboratories) at 18°C and purified using chromatographic procedures as described previously (40). The following are the IMAGE IDs of the Pax cDNA clones used in the study: Pax2 (IMAGE clone ID: 40142579), Pax3 (IMAGE clone ID: 6518115), Pax6 (IMAGE clone ID: 4504106), Pax8 (IMAGE clone ID: 4239835) and Pax9 (IMAGE clone ID: 3707718). The paired domain sequences were GATEWAY BP cloned (Life Technologies) from their respective cDNA clones into the pDONR221 vector using primers with attB1 sites (Supplementary Table S1). The resulting pENTR constructs were verified by sequencing and recombined into the pETG40A and pETG20A destination vectors using the GATEWAY™ technology (Life Technologies). Recombinant Pax proteins were expressed and purified using a previously published protocol (41). Expression vectors with point mutations in Sox2-long (D123G) and Pax6 (R44Q and G36R) were generated using the QuikChange XL mutagenesis kit (Stratagene) according to manufacturer's instructions (Supplementary Table S1). The incorporation of point mutations was verified by sequencing the expression plasmid.

### DNA elements used

DNA sequences (Supplementary Table S2) labeled with 5-carboxyfluorescein (FAM) or Cy5 were employed in the study. Single-stranded oligos were purchased from Sigma Proligo and cognate double-stranded DNA elements were made by annealing the oligos in annealing buffer (20 mM Tris–HCl, pH 8.0; 50 mM MgCl_2_; 50 mM KCl), heated to 95°C for 5 min and subsequently ramping down at 1°C/min to 4°C in a polymerase chain reaction block. To study the effect of spacing between the *sox* and *pax* motifs, the following *DC5* sequences with different spacing were used: -1 and -2 with reduced spacing and +1, +2, +3, +4 and +5 with increased spacing between the *sox* and *pax* half-sites. For affinity measurements, a previously crystallized DNA element (PDB ID: 6PAX) was used.

### Electrophoretic mobility shift assays

Electrophoretic mobility shift assays (EMSAs) were performed with 5′-FAM or 5′-Cy5-labeled cognate double-stranded DNA elements (Supplementary Table S2). The binding buffer contains 10 mM Tris–HCl pH 8.0, 0.1 mg/ml bovine serum albumin, 50 μM ZnCl_2_, 100 mM KCl, 10% Ultrapure Glycerol, 0.10% IGEPAL CA-630 (octylphenoxypolyethoxyethanol) and 2 mM β-mercaptoethanol. For the differential assembly studies, typical binding reactions contain concentrations 100–300 nM of double-stranded DNA (>> *K*_d_) with varying concentrations of Sox and Pax proteins. Protein concentrations were adjusted such that an equilibrium is reached and the four microstates are visible as distinctive bands on EMSA gels to allow reproducible quantification. All binding reactions were incubated for 1–2 h in the dark. Samples were loaded onto 1X Tris Glycine-PAGE gels (25 mM Tris pH 8.3; 192 mM glycine) and electrophoresed at 200 or 300V for 30–120 min at 4°C. The gel was imaged using a Typhoon 9140 phosphor imaging scanner or a Typhoon FLA-7000 PhosphorImager (FUJIFILM) and the fluorescent intensities of the free and bound DNA were quantified using the ImageQuantz TL software. Cooperativity factors were calculated using the formula ω = (f_dimer_*f_freeDNA_)/(f_monomer1_*f_monomer2_) where f_dimer_ is the fractional contribution of dimeric protein complexes on DNA (Sox/Pax/DNA), f_monomer1_ and f_monomer2_ of monomeric (Sox/DNA or Pax/DNA) protein–DNA complexes and f_freeDNA_ the fractional contribution of unbound DNA in EMSA gel lanes. The derivation of the formula has been described previously (16,42) and was further discussed in (43).

### Homology modeling of Sox2–Pax6

The models of the Sox2–Pax6–DNA ternary complexes were build by superimposing B-DNA fragments corresponding to the *DC5, N3* and *DC5con* sequences on the N-terminus and C-terminus of the structures of the Sox2/DNA and Pax6/DNA complexes, 1GT0 (13) and 6PAX (30), respectively. In addition, the DNA molecules at the C-terminus of the 1GT0 structure and at the N-terminus of the 6PAX structure were superimposed, so as to reflect the spacing between the Sox and Pax half-sites observed in the *DC5, N3* and *DC5con* sequences. The sequence of the new DNA formed by concatenating the four DNA fragments and removing the overlapping bases was mutated to the correct *DC5, N3*, and *DC5con* sequences using chimera (https://www.cgl.ucsf.edu/chimera/). The energy of the resulting models of the ternary complexes was minimized in AMBER (www.ambermd.org) using a stepwise procedure in which restraints on the base pair geometries were gradually removed.

### Bioinformatics analysis

Human FANTOM5 CAGE data (http://fantom.gsc.riken.jp/5/) were downloaded and condensed into a single table after averaging some duplicates and only the highest expressed promoter was kept for a given cell type. The clustered heatmap was produced after row normalization using glbase (44). ChIP-Seq peaks for PAX6 in human NSCs (45) or SOX2 neural progenitor cells (NPCs) (46) were intersected. Motifs were created manually taking EMSA data into account and co-bound SOX2/PAX6 sites were searched for motif ‘words’ using glbase (44). To produce genome plots using IGV, reads were re-mapped to the human genome (hg19).

### Circular dichroism spectroscopy

The circular dichroism (CD) spectra were recorded on an Chirascan CD Spectrometer (Applied Photophysics Ltd) using a strain-free 10 mm x 1.0 mm rectangular cuvette using the following parameters: bandwidth, 1 nm; spectral range, 230–360 nm; step-size, 1 nm; time-pep-point, 0.5 s. In total, 1.6 μM dsDNA and 5 μM Pax6-PRD were mixed in 50 mM phosphate buffer, pH 8.0. Three repeat measurements were averaged.

### Nuclear magnetic resonance experiments, data acquisition and processing

Nuclear magnetic resonance (NMR) experiments were performed using Bruker AVANCE II 600 and 700 MHz NMR spectrometers equipped with a standard TCI (Triple resonance cryoprobe) cryoprobe. The spectra were collected at 298 K. A series of 2D [^15^N, ^1^H]-TROSY experiments was utilized to monitor the chemical shift perturbations (CSP) of the ^15^N, ^1^H spins. The chemical shifts were referenced directly (^1^H) relative to 4,4-dimethyl-4-silapentane-1-sulfonic acid (DSS). The spectral analysis was performed using CARA (47). For NMR experiments, a 31bp *DC5* element was used with the following forward and complementary reverse strand sequence respectively (*DC5*_f: 5’-TTCATTGTTGTTGCTCACCTACCATGGATCC-3’; *DC*5_r: 5’-GGATCCATGGTAGGTGAGCAACAACAATGAA -3’).The 31bp *DC5* DNA element was dissolved in the following NMR buffer (50 mM K_2_HPO_4_/KH_2_PO_4_, 100 mM NaCl buffer, pH 7.0). The buffer components are kept consistent for all NMR experiments unless otherwise stated. The pH of the solutions was adjusted to 7.0 using NaOH. The final concentration of the *DC5* stock solution was determined using NanoDrop at 260 nm. We have previously recorded 3D spectra of ^1^H, C^13^ and N^15^ labeled Sox2 (109 amino acid long) and assigned residues to the majority of resonances (40). The N^15^ labeled Sox2-HMG was added carefully to the DNA element (31-mer) in a stepwise manner to achieve final molar ratios of Sox2/DNA of 0.25, 0.5, 0.75 and 1.0, with the final DNA concentration of 0.2 mM. The 1D spectrum of the 31mer-DNA alone was used as a reference to observe perturbations inflicted by the binding of proteins to the DNA. A 2D [^15^N, ^1^H]-TROSY spectrum was collected at each titration point. The weighted CSP for backbone ^15^N and ^1^H_N_ resonances were calculated by the equation (48): Δδ = [(Δδ_HN_)^2^ + (0.1Δδ_N_)^2^]^0.5^.

## RESULTS

### Pax motif degeneracy is necessary for the cooperativity of Sox2 and Pax6

**Figure 2.**
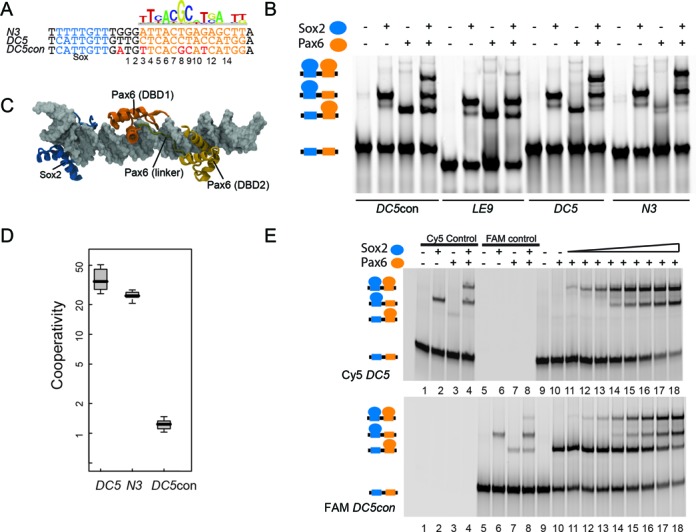
Pax motif degeneracy is necessary for cooperative ternary complex assembly. (**A**) A SELEX-derived Pax6 PWM (50) was downloaded from JASPAR and is displayed above *DC5* (21) and *N3* (36) enhancer sequences. The residues introduced into the *DC5con* element to generate a *pax* site resembling the SELEX consensus are shown in red. (**B**) EMSAs to monitor the formation of ternary Sox2–Pax6–DNA complexes on *DC5con, LE9, DC5* and *N3* elements. The cartoon on the left depicts different complexes obtained during EMSA. (**C**) A structural model of Sox2–Pax6–DNA complex is shown. The DNA is shown as gray surface. The Sox2-HMG derived from PDB-ID 1GT0(13)is shown in blue and the Pax6-PRD (PDB-ID 6PAX (30)) in orange. Pax6-PRD subdomains (DBD1 and DBD2) and the linker are marked. (**D**) Cooperativity factors are calculated after quantifying the four possible microstates seen on EMSA gels (free DNA, Sox2.DNA, Pax6.DNA and Sox2.Pax6.DNA) using established procedures (16) and are shown as boxplots using data from 9–13 replicate experiments. Values for the *LE9* enhancer could not be reliably determined as the ternary complex bands were barely detectable. (**E**) EMSAs were performed by simultaneously incubating Sox2 and Pax6 with differently labeled *DC5* and *DC5con* DNA. Gels are shown after scanning using Cy5 (670 nm band-pass emission filter) or FAM (520nm band-pass emission filter) settings on a Typhoon phosphorimager. In total, 300 nM of each Cy5-*DC5* and FAM-*DC5con* were used along with 100 nM Pax6 (FAM and cy5 control, lanes 3,4,7,8) or 300 nM Pax6 (lanes 10–18). Moreover, 100 nM Sox2–109aa was used in the Cy5 and FAM control reactions (lanes 2,4,6,8) or with increasing concentrations from 25 to 600 nM (lanes 11–18).

Sox2 and Pax6 are co-expressed in a number of human cell types, namely fetal eyes, NSCs, cerebellum and hippocampus (Figure 1A). A large number of other Sox and Pax family members are co-expressed in other cell types (Figure 1B). Given that Sox2 and Pax6 have been shown to act as ‘master regulators’ of pluripotency and neurogenesis, we surmised that Sox–Pax cooperativity is key for cell fate decisions. We previously set up quantitative EMSAs to estimate cooperativity factors (ω), i.e. the ratios of the equilibrium binding constants of dimeric versus monomeric complexes of TFs binding to composite DNA enhancer elements (16,42). Three minimal native sequences extracted from the *DC5* (24), *N3* (36) and *LE9* (49) enhancer elements were chosen to study the interaction between the Sox2-HMG and Pax6-PRD domains (henceforth termed Sox2 and Pax6). In addition, we used a modified sequence of the *DC5* element, *DC5con* (24) where the native low-affinity *pax* half-site is replaced with a high-affinity half-site as determined by SELEX (32,50) (Figure 2A). In agreement with previous studies (24), Sox2 and Pax6 were found to form ternary complexes on both the *DC5* and *N3* enhancer elements (Figure 2B). By contrast, using the *LE9* enhancer (49), we observed competitive binding (Figure 2B). When the *pax* half-site of the wild-type *DC5* element was replaced with *DC5con*, ternary complex formation was markedly weakened as evident from the observation that Sox2–DNA and Pax6–DNA complexes are still prominently visible in addition to the ternary complex suggesting ineffective complex formation (Figure 2B). To understand the structural basis for the cooperativity, we generated structural models of Sox2–Pax6 complexes on *DC5con, DC5* and *N3* enhancer sequences (Figure 2C). The models show that Sox2 and Pax6 do not interact directly through the globular core of their DBDs. However, they may form some protein–protein interactions through the C-terminal tail of Sox2 and communicate through DNA-mediated allostery. Indeed, when we calculated the cooperativity factor, we obtained values between 20 and 50 for Sox2–Pax6 cooperation on *DC5* and *N3* elements, respectively (Figure 2D). However, on the *DC5con* element, we measured additive binding (ω ∼ 1), demonstrating that degenerated *pax* half-sites as found in the native *DC5* and *N3* elements are essential for the cooperative interaction of Sox2 and Pax6. Next we incubated Sox2 and Pax6 simultaneously with Cy5-labeled *DC5* and FAM-labeled *DC5con* to evaluate the distribution of Sox2 and Pax6 on alternative target sites. Binary Pax6–DNA complexes are detectable at low Pax6 concentrations on the high-affinity *DC5con* element. By contrast, ternary Sox2–Pax6–DNA complexes predominate on the high-cooperativity *DC5* element and binary Pax6–DNA complexes are barely visible (Figure 2E). This suggests that in a cellular environment where large numbers of DNA motifs compete for TF binding, Pax6 is effectively recruited to its consensus binding site. However, activation-competent ternary Sox2–Pax6 complexes are more effectively recruited to composite motifs with cryptic *DC5*-like *pax* half-sites.

### The composite Sox2–Pax6-binding site is compact and rigidly spaced

**Figure 3.**
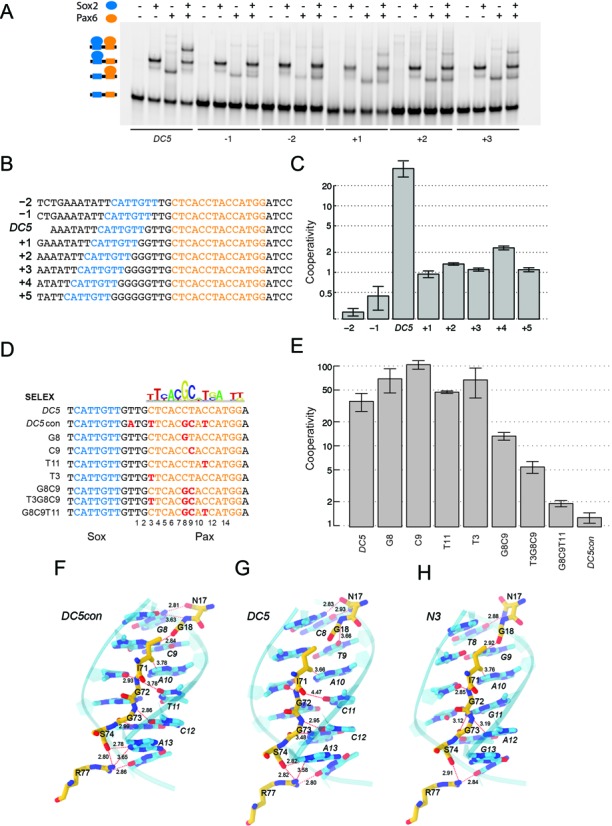
Effect of motif spacing and sequence features on Sox2–Pax6 cooperativity. (**A,B**) EMSAs using *DC5* enhancer elements with systematically altered spacing between *sox* (blue) and *pax* (orange) binding elements. (**C**) Cooperativity factor measurements revealed a drop in the cooperativity by at least an order of magnitude for all artificial spacers as compared to the native element. (**D**) *DC5* sequences were sequentially converted into *DC5con*-like sequences corresponding to the Pax6 consensus and cooperativity factors were determined (**E**). Whiskers in barplots indicate standard deviations. (**F**) Interactions between Asn14 and Gly15 and *DC5con*-like sequence as seen in the 6pax crystal structure and the *DC5con* model and perturbations of these interactions when Pax6 is bound to *DC5* (**G**) or *N3* (**H**) sequences.

Next we studied the effect of inter-motif distances on the efficiency for Sox2–Pax6 dimerization. The 2, 4 and 10 base pair spacers have previously been found to abrogate Sox2–Pax6 cooperation on the *DC5* element (24). Here we extended these studies by interrogating the effect of motif spacing from -2 to 5 base pairs at 1-bp intervals (Figure 3A and B). We observed that even subtle changes to the spacing between the *sox* and *pax* half-sites had profound effects on complex formation (Figure 3A–C). Ternary complexes are barely visible and cooperativity factors turn negative upon reducing the spacing between the motifs by 1 and 2 bp. This indicates competitive binding possibly due to steric hindrance when the proteins are brought too close to each other. Upon increasing the spacing (+1,+2,+3 & +5), the cooperativity factor markedly decreases (∼30-fold) reminiscent of the additive binding seen on the *DC5con* element (Figure 3B and C). The +4bp spacer is an exception as it retains a weak albeit still strongly reduced cooperativity as compared to the other non-native spacers (Figure 3C). Collectively, these experiments highlight that precise motif juxtaposition is necessary for the formation of a cooperative Sox2–Pax6–DNA complex. Even small perturbations to the enhancer architecture lead to drastic reductions in ternary complex assembly, suggesting rigid spatial constraints for the assembly of the complex.

### Nucleotide switches act in concert to enable cooperative Sox2–Pax6 interaction

**Figure 4.**
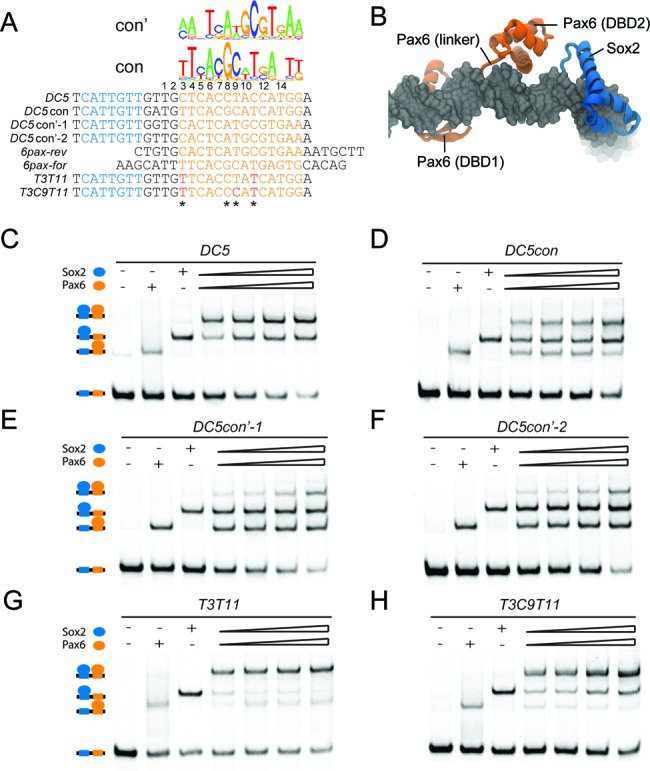
Sox2 and Pax6 do not cooperate on *DC5con’* elements encoding a flipped *pax* consensus. (**A**) The sequence logo of forward and reverse *pax* consensus motifs is aligned with sequences of *DC5, DC5con, DC5con’* and forward and reverse sequences co-crystallized with Pax6 (PDB-ID 6pax). Switch nucleotides (Figure 3) are marked with asterisks. The nucleotides 3, 9 and 11 found to be part of the ‘cooperativity switch’ are identical between *DC5* and *DC5con’*. (**B**) Structural model of a Sox2/Pax6 complex on the *DC5con’* sequence with Pax6 in a hypothetical flipped conformation. EMSAs to study the cooperativity of Sox2 and Pax6 on *DC5* (**C**), *DC5con* (**D**), *DC5con’-1* (**E**), *DC5con’-2* (**F**) and mutations to make the *DC5* sequence more closely match the *DC5con* sequence T3T11 (**G**) and T3C9T11 (**H**).

Next we aimed to identify the sequence features that convert the high-cooperativity *DC5* element that facilitates dimer formation of Sox2 and Pax6, into a site detrimental to their co-recruitment. The pwm (position weight matrix) of the ideal Pax6 motif (JASPAR ID: MA0069.1) derived from *SELEX* assays (50) identifies four nucleotide positions with high information content that deviate from the degenerate *pax* half-site in the native *DC5* and *N3* elements (Figure 3D). These positions will be referred to as T3, G8, C9 and T11 (Figure 3D). In order to dissect the role of these four key nucleotide positions in the *DC5* element, we sequentially converted the degenerate *pax* half-site in the native *DC5* element to resemble the high-affinity *DC5con pax* half-site (Figure 3D). Interestingly, point mutations to any of the four key nucleotide positions in the *DC5* element did not decrease cooperative binding (Figure 3E). In fact, some point mutations even led to an elevation of the cooperativity, including the G8 and C9 positions (Figure 3E). However, a G8C9 di-nucleotide mutation substantially lowered the cooperative binding (Figure 3E). The tri-nucleotide mutations T3G8C9 and G8C9T11 further enhanced this effect and successively approached a cooperativity reminiscent of *DC5con* (Figures 2C and 3E). This suggests that the nucleotide positions do not linearly contribute to the complex formation but are interdependent. Our models of the three ternary complexes revealed that the interactions between the Pax6-PRD around nucleotides G8, C9 and T11 were found to be most optimal in *DC5con* (Figure 3F). The less optimal configuration of Pax6 bound to the *DC5* (Figure 3G) and *N3* (Figure 3H) sequences may be further improved by small reorientations of the Pax DBDs that may lead to configurations enhancing cooperativity with Sox2. Such configurations may also be triggered by changes in DNA structure induced by the presence of Sox2. However, it cannot be ruled out that, the *DC5* and *N3* sequences may induce an assembly of Pax6 in a conformation completely different from the one in our models, which resemble that in the structure of a previously solved Pax6/DNA complex (PDB: 6PAX) (30). Alternatively, the orientation of Pax6 could change to accommodate the *DC5* sequence. Indeed, when we inspected the reverse-complement of the *pax* consensus sequence (con’) and of the consensus sequence co-crystallized with Pax6 (30) (*6pax-rev*), we observed a good agreement with *DC5* (Figure 4A). In particular, nucleotides 3, 9 and 11 found to be critical for the ‘cooperativity switch’ (Figure 3) align perfectly between *DC5* and *6pax-rev*. We therefore decided to test if the *DC5* element encodes a cryptic pax consensus and mediates cooperative binding through a changed orientation of Pax6. To this end, we constructed *DC5con’-1* and *DC5con’-2* sequences containing a reverse complement of the sequence found in the 6pax crystal structure spaced appropriately so that the key nucleotides 3, 9 and 11 align with wild-type *DC5* (Figure 4A). Structural modeling (refer Material and methods, Homology modelling section) suggests that Sox2 and Pax6 could co-bind such sequences (Figure 4B). However, EMSAs revealed that complex formation is inefficient on both tested *DC5con’* sequences and monomeric complexes predominate (Figure 4C–F). Moreover, when the three nucleotides 3, 9 and 11 that match *DC5* in *DC5con’* but not in *DC5con* were mutated in *DC5*, the cooperativity remained high (Figure 4G and H). Collectively, these experiments suggest that Pax6 is unlikely to bind *DC5* in a ‘flipped’ conformation (Figure 4B).

### Cooperative binding to specific enhancer sequences is a conserved trait in the Sox and Pax protein families

**Figure 5.**
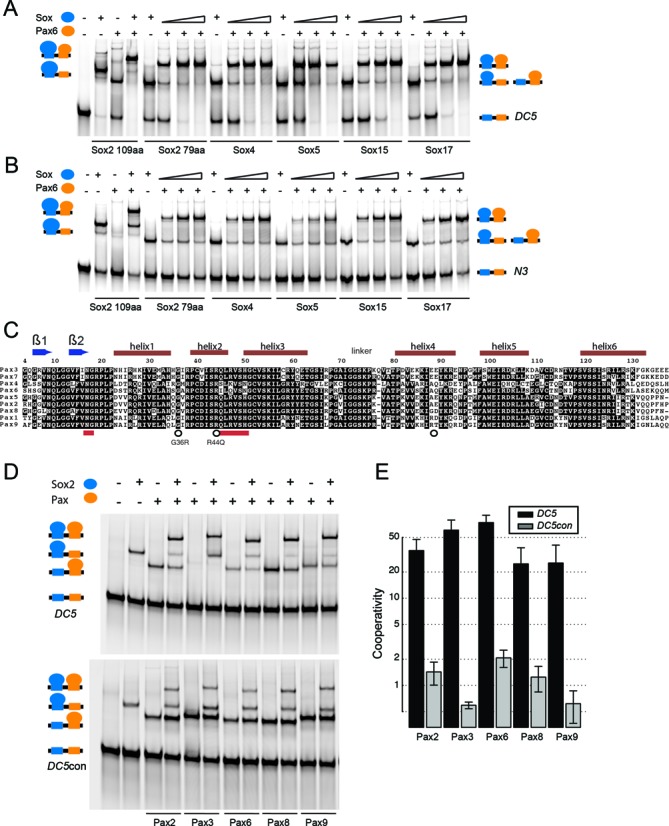
The potential to cooperate is conserved in the Sox and Pax TF families. Formation of ternary complexes between Pax6 and long (109aa) and short (79aa) versions of the Sox2-HMG and paralogous mouse Sox HMGs (79aa) of Sox4, Sox5, Sox17 and Sox15 on (**A**) *DC5* and (**B**) *N3* elements. Note that 79 amino acid Sox-HMG versions co-migrate with the Pax6-PRD bound to DNA, whereas the 109aa version migrates as distinctive band. (**C**) Multiple sequence alignment of mouse Pax paralogs marking secondary structure elements above the alignment. Numbering corresponds to human PAX6 uniprot-ID P26367 for easier reference to eye diseases mutations. Red bars mark the Asn17 and Gly18 di-peptide binding to the di-nucleotide switch and residues previously shown to provide discriminatory sequence recognition between Pax6 and Pax5 (32). (**D** and **E**) The Sox2-HMG (109aa) was assessed for its propensity to cooperate with Pax family members Pax2, Pax3, Pax6, Pax8 and Pax9.

We previously studied cooperative binding of Sox and Oct protein families to identify signature enhancer sequences. We observed a partner code with distinctive Sox-Oct combinations binding specific composite *sox-oct* DNA motifs (16). Selective binding relies on certain amino acids of the Sox HMG and interchanging these residues between Sox factors swaps their ability to dimerize with Oct4 on specific composite motifs and to direct cell fate decisions during pluripotency or endoderm induction (14–15,51). To test whether a similar partner code exists for Sox–Pax interactions, we purified representative members of the Sox and Pax families and conducted quantitative cooperative measurements. First we asked whether Pax6 selectively cooperates with Sox2 or if it also teams up with other Sox factors. To this end, we selected members from several subgroups of the 20-member Sox family. Specifically, we chose Sox4 of the SoxC group, Sox5 of the SoxD group, Sox17 of the SoxF group and Sox15 of the SoxG group and tested them alongside the SoxB1 group member Sox2. We found that Sox2, Sox4, Sox5, Sox15 and Sox17 cooperatively bound with Pax6 to *DC5* and *N3* enhancer elements in an indistinguishable fashion (Figure 5A and B). Next we conducted inverse experiments and compared the cooperative binding of Sox2 with Pax2, Pax3, Pax5, Pax6, Pax8 and Pax9 to the *DC5* sequence. There are some sequence variations within the PRD paralogs, some of which were previously found to be critical for discriminative DNA binding (32) (Figure 5C). Nevertheless, we found that all Pax family members can cooperatively pair-off with Sox2 on the *DC5* element, whereas the cooperativity is abolished on the *DC5con* element (Figure 5D and E). This suggests that all Pax PRDs possess biochemical features conducive for the cooperation with Sox2 on specific enhancers with cryptic Pax6-binding sequences. Therefore, cooperativity on *DC5*-like sequences is a biochemical trait conserved within Sox and Pax TF families. As Sox and Pax paralogs are co-expressed in diverse cell types (Figure 1B), it can be envisaged that there are ample opportunities for members of the two families to interact. However, the propensity to cooperatively recognize *DC5*-like sequence *in vitro* does not imperatively suggest that all co-expressed Sox/Pax pairs combinatorially regulate gene expression and further studies are necessary to establish new functional Sox/Pax partnerships. First, it has to be assessed whether co-expressed Sox/Pax pairs indeed co-bind enhancer sequences. Second, it has to be tested whether the binding event leads to the transactivation of a target gene.

### DNA binding of clinically relevant Sox2 and Pax6 mutant proteins

**Figure 6.**
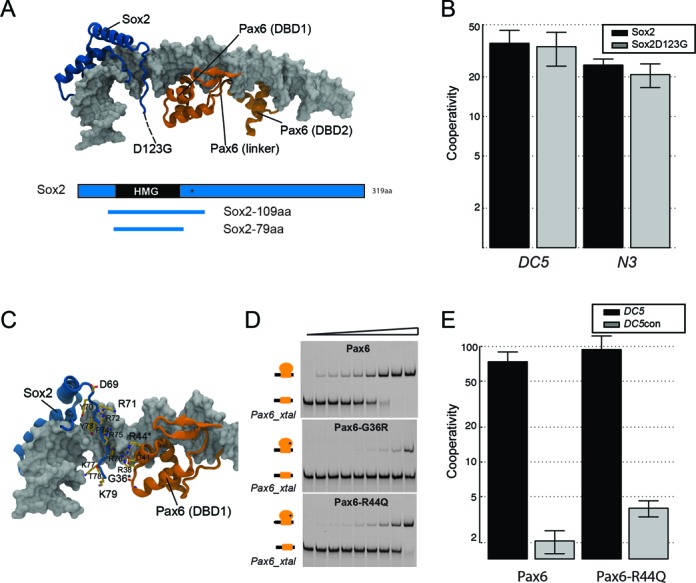
Eye disease mutations and Sox2–Pax6 interactions. (**A**) Structural model of a hypothetical Sox2–Pax6 complex. The D123G mutation (57) maps to a region in the C-terminal tail just off the portion visible in the crystal structure (indicated with arrow and dashed line). A domain plot indicating the Sox2 constructs used for cooperativity measurements are shown below. (**B**) A barplot comparing cooperativity factors for the interaction of Sox2 versus Sox2D123G with Pax6 on *DC5* and *N3* DNA elements. Whiskers indicate standard deviations from seven to nine measurements. (**C**) Location of two Pax6 mutations R44Q and G36R within the Pax6 PRD. (**D**) The missense mutations lower the affinity for DNA as compared to the wild-type PRD on a consensus Pax6-binding element (Pax6_xtal, Supplementary Table S1). (**E**) Cooperativity measurements for Pax6 and Pax6R44Q on *DC5* and *DC5*con elements show that the R44Q mutation does not abolish cooperative complex formation. Whiskers indicate standard deviations from five to six measurements.

Several eye diseases including blindness and aniridia are caused by heterozygous Pax6 mutations, several of which map to the paired domain (52–56). Likewise, Sox2 mutations were recently reported to be associated with visual disorders (57,58). As the biochemical consequences that lead to disease are not understood for most of those mutations, we tested whether DNA binding or protein-partnerships are perturbed. One selected mutation was found in a four-generation family proband in Australia that has bilateral anophthalmia and several milder visual system anomalies like typical optic fissure coloboma (57). A novel D123G mutation in the C-terminal tail following the Sox2 HMG domain was detected in this family that was attributed to anophthalmia (57). Since D123G maps to a region near the hypothetical binding interface between Sox2 and Pax6 (Figure 6A), we examined whether this mutation alters the propensity to a form ternary complex. However, we could not detect any changes in complex formation compared to the wild-type Sox2 (Figure 6B). Similarly, co-binding with Oct4 on the *Fgf4* enhancer element was also not altered for the Sox2D123G mutation (Supplementary Figure S1). Next we selected the Pax6 missense mutations G36R and R44Q previously detected in aniridia patients (59,60). In our structural models, those mutations are in proximity to the putative Sox–Pax contact interface (Figure 6C). We therefore wanted to examine whether these two mutations could alter cooperativity with Sox2. We observed that G36R and R44Q markedly reduced affinity for DNA binding (Figure 6D). However, Pax6R44Q did not affect the cooperativity with Sox2 on the *DC5* element (Figure 6E). The cooperativity factor for Pax6G36R could not be determined due to the low overall affinity for DNA. Therefore, diminished DNA binding of the Pax6 protein likely causes the development of aniridia in Pax6R44Q and Pax6G36R patients. Consistently, in co-crystal structures of the highly homologous *Drosophila* Prd protein bound to DNA it can be seen that the residue homologous to R44Q is in contact with the sugar phosphate backbone of DNA (29).

### NMR and modeling imply a DNA-mediated allosteric mechanism for Sox2–Pax6 interaction

**Figure 7.**
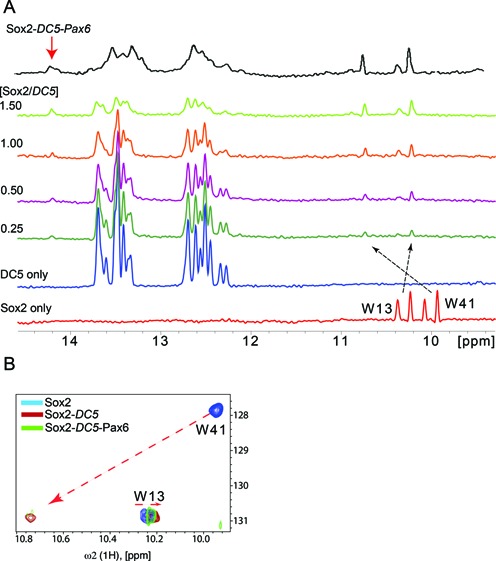
Assembling a stable ternary complex for NMR studies. (**A**) 1D ^1^H spectra with increasing stoichiometry of Sox2-HMG in complex with *DC5*, from bottom to top (Sox2 to *DC5* ratios at 0.25, 0.5, 1.0, 1.5) as well as a Sox2–Pax6–*DC5* ternary complex. (**B**) The tryptophan indole spectral region of 2D [^15^N,^1^H]-TROSY spectra. The chemical shift changes in the tryptophan Ne1/He1 side chain groups are indicated by arrows for Sox2 only (blue) in the presence of *DC5* (red) and the ternary Sox2–*DC5*–Pax6 complex (green).

The mechanism of the cooperative recognition of *DC5* DNA by Sox2 and Pax6 cannot be straightforwardly modeled based on available experimental structures. Our biochemical assays and structural models suggest that in particular the Pax6-PRD might adopt different conformations compared to the configuration seen when bound to its high-affinity element (29,30). Such conformational versatility has been described for the POU family that also contains a bi-partite DBD. POU members were found to adopt strikingly different tertiary structure and altered stoichiometry depending on the sequence of the bound DNA element (61–63). To define the protein–protein interaction interface between Sox2 and Pax6, we performed NMR studies. First, we measured 1D ^1^H spectra of Sox2 alone, *DC5*, Sox2-*DC5* and ternary Sox2–Pax6–*DC5* complexes (Figure 7A). *DC5* in complex with Sox2 showed significantly more dispersed imino proton resonances at the region between 12 and 14.5 ppm indicating bending of the DNA induced by Sox2 (Figure 7A). In addition, we observed significant chemical shifts and differential line broadening of the ^1^H doublet signals from the indole groups of the Sox2 tryptophan residues W13 and W41 in the 10–11 ppm region upon DNA addition, indicating a significant increase in molecular weight and effective complex formation. Addition of Pax6 to the Sox2–*DC5* binary complex enhances differential line broadening in the ^1^H doublets of W13 and W41, indicating further molecular weight increase upon formation of the ternary complex (Figure 7A). However, binding of Pax6 to the Sox2–*DC5* complex results only in relatively minor shifts of both Sox2 tryptophan ^15^N and ^1^H resonances (Figure 7B). Yet, the ^1^H resonance corresponding to W41 (ϵH) is observed to attenuate considerably while W13 (ϵH) remains stable (Figure 7A and B). These results suggest that incorporation of Pax6 into a ternary complex might affect the conformational dynamics of Sox2 and possibly affects how Sox2 bends DNA.

**Figure 8.**
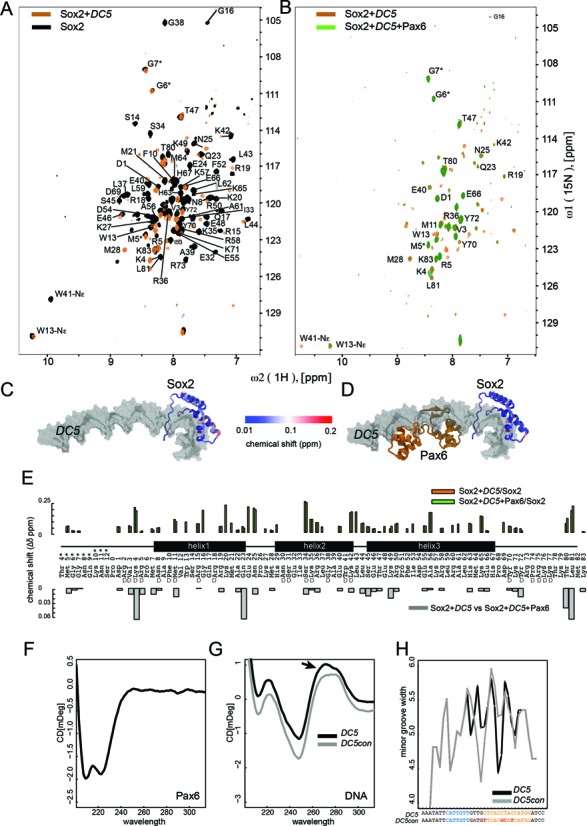
Sox2–*DC5* and Sox2–Pax6–*DC5* interactions analyzed by [^15^N,^1^H]-TROSY. (**A**) Superposition of 2D [^15^N,^1^H]-TROSY spectra of free Sox2 (black) and Sox2–*DC5* (orange) and (**B**) Sox2–*DC5* (orange) and Sox2–Pax6–*DC5* (green) on the right panel. The spectra are acquired at the stoichiometric ratios of 1:1.2 (Sox2–*DC5*) and 1:1.2:1 (Sox2–*DC5*–PAX6). (**C** and **D**) The Sox2 CSP weighted as Δδ = [(Δδ^1^H^N^)^2^ + (0.1ΔδN)^2^]^−1/2^ are mapped onto the Sox2-HMG in Sox2–*DC5* (C) and Sox2–Pax6–*DC5* models (D). (**E**) CSP of Sox2 in binary (orange) and ternary (green) complexes relative to free Sox2 versus the Sox2 amino acid sequence (numbered according to Sox2: PDB 1GT0 (13)). ‘D’ denotes DNA-binding residues. The lower panel shows weighted Sox2 CSP of the binary versus ternary complexes. CD spectra recorded of Pax6 (**F**) and DNA (**G**). The arrow highlights the slightly shifted peak at 280 nm. (**H**) The width of the minor grooves in *DC5* and *DC5con* as estimated by GBshape (64) (http://rohsdb.cmb.usc.edu/GBshape/).

Comparative ^15^N/^1^H Sox2 spectra before and after *DC5* addition revealed CSP and conformational-exchange induced line broadening for many peaks (Figure 8A and B). By mapping CSPs to our models of Sox2/DNA and Sox2/Pax6/DNA complexes (13), we found that residues undergoing CSPs are not restricted to the DNA contact interface, suggesting global structural rearrangements of Sox2 upon DNA binding (Figure 8C and D). The largest CSPs (> 0.1 ppm) observed are for K4, G16, K20, E24, N25, S34, K42, L43, A56 and K65 (Figure 8E). Residues that experience line broadening are predominantly located in the hydrophobic core of the protein. When Pax6 is added to form the Sox2–Pax6–*DC5* ternary complex, only a few additional CSPs were observed in Sox2 (Figure 8B, D and E: K4, Q23, T80, L81). Yet, Pax6 addition leads to a considerable attenuation of peak intensity in the region of the major wing (helices 1 and 2), indicating that Pax6 may primarily affect the dynamics and stability of the Sox2–*DC5* interactions rather than directly binding to Sox2 and altering its conformation. Notable exceptions are R15 and R19 that experience an increase in the peak intensity after Pax6 addition potentially indicating more favorable electrostatic interactions for these residues in the ternary complex. CD measurements showed that the DNA peak at 280 nm is slightly shifted in *DC5* as compared to *DC5con* DNA (Figure 8F and G). Likewise *in silico* predictions (GBShape (64) ) of the minor groove width suggest an altered pattern of groove compressions over the *pax* half-site (Figure 8H). Those structural differences could affect the propensity of the respective DNA sequences to mediate the co-recruitment of Sox2 and Pax6. Collectively, our data suggest that Sox2–Pax6 cooperation on *DC5*-like sequences is not mediated by complementary protein–protein interactions but rather by an allosteric, DNA-mediated mechanism that relies on a subtly adjusted DNA structure in agreement with our models of the ternary complexes. Collectively, our data suggest that, *DC5*-like sequences support a predominantly DNA-mediated mechanism of cooperativity, but *DC5con*-like sequences do not.

### Identification of cryptic SOX2/PAX6 cooperativity loci in human NSC enhancers

**Figure 9.**
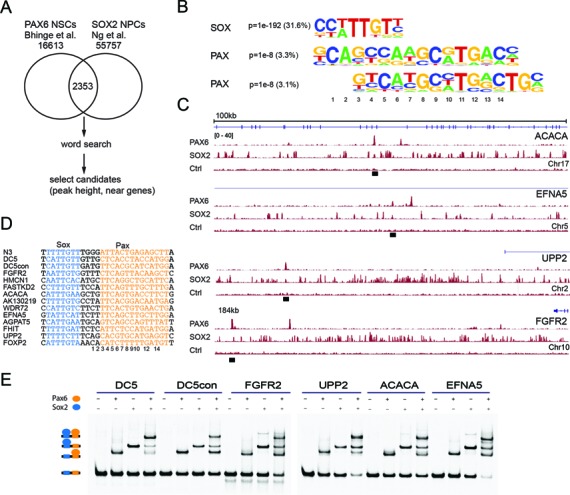
Identification of loci supporting Sox2/Pax6 dimerization during human neurogenesis. (**A**) Scheme illustrating the selection of candidate *DC5*-like sequences bound by PAX6 and SOX2 in human cells. (**B**) *De novo* motifs recovered using homer (http://homer.salk.edu/homer/chipseq/) in SOX2/PAX6 co-bound loci. (**C**) Genome plots spanning 100 kb (*ACACA:* acetyl-CoA carboxylase Alpha*, EFNA5:* ephrin-A5*, UPP2 -* uridine phosphorylase 2) or 184 kb (*FGFR2:* fibroblast growth factor receptor 2) are shown for selected candidate loci predicted to recruit SOX2/PAX6 dimers in a cooperative fashion. Black bars indicate binding sites with *DC*5-like sequences used for EMSA validation. Binding site definitions were used as reported in the original publications (45,46). (**D**) Sequences of novel *DC5*-like composite motifs short-listed for EMSA validation. (**E**) EMSAs showing effectively formed SOX2/PAX6 dimers on newly identified NSC enhancer sequences.

The chicken *DC5* and *N3* sequences support cooperative and functionally important Sox2/Pax6 interactions. However, the cooperative interaction of SOX2 and PAX6 on related sequences has so far not been reported for human cells. We hypothesized that SOX2 and PAX6 also cooperate on *DC5*-like sequences during human neurogenesis. To test this hypothesis, we took advantage of recently published ChIPseq studies of PAX6 in human NSCs (referred to as neuroepithelial cells by the authors) (45) and SOX2 ChIPseq data from NPCs (46). While both cell types are not identical, they constitute the most closely related human cell types for which ChIPseq data are presently available. After intersection, we obtained 2353 regions co-bound by PAX6 and SOX2 (Figure 9A). *De novo* motif searches revealed that more than 30% of the shared loci contain consensus *sox* motifs (Figure 9B). However, known *pax* motifs are only present in ∼3% of the putative NSC enhancers, suggesting that the vast majority of Pax6-targeted sites does not rely on high-affinity *pax* motifs *in vivo*. As only the *N3* and *DC5* sequences are known to support Sox2/Pax6 cooperation, there is no position-weight matrix or related binding models for capturing cryptic *pax* half-sites as observed in the *DC5 or N3* enhancer. We therefore manually constructed degenerate *DC5/N3*-like consensus motif ‘words’ based on our EMSA results. We used these motifs to search the 2353 NSC enhancers and detected 20 *DC5*-like sequences, most of which exhibit a strong ChIPseq signal indicative of effective Pax6 binding (Figure 9C). We selected eight of those sequences for EMSA validation (Figure 9D). All tested sequences showed ternary Sox2–Pax6–DNA complexes illustrating that high-affinity *pax* motifs are not required for complex formation (Figure 9E). Moreover, several enhancer sequences supported the cooperative recruitment of Sox2 and Pax6 reminiscent of *DC5* and *N3* enhancer elements (Figure 9E). In conclusion, the partnership between SOX2 and PAX6 on cryptic *DC5*-like sequences also occurs during human neurogenesis and possibly underlies the combinatorial action of those TFs during cell fate determination.

## DISCUSSION

The present work takes advantage of the seminal studies by Kamachi, Kondoh and co-workers revealing that Sox2 and Pax6 cooperate on the 30 bp *DC5* core enhancer to activate the δ-*crystallin* gene in chicken eyes (21–22,24). Their work suggested a striking example of TF association on composite DNA elements despite the lack of a high-affinity consensus sequence for Pax6. Therefore, the *DC5* eye enhancer provided a paradigm, highlighting the intricacies of how TFs pair off to read the regulatory code underlying developmental programs (21,24). The functional studies and the intricate behavior in qualitative binding assays encouraged us to quantitatively dissect Sox2 and Pax6 cooperativity and the molecular basis for this partnership. Collectively, we found that (i) cooperativity critically relies on degenerate *pax* sites and that consensus sites abrogate ternary complex formation; (ii) highly constrained spacing arrangements of *sox* and *pax* half-sites are critical for cooperative binding; (iii) key nucleotide switch positions in the degenerate *pax* half-sites act in concert to mediate cooperative assembly of Sox2 and Pax6; (iv) the mechanism of cooperative binding is evolutionary conserved within Sox and Pax families and, finally, (v) direct protein–protein interactions between Sox2 and Pax6 are sparse if present at all. Overall, these results suggest that stable Sox–Pax interactions rely on specific DNA sequences and not complementary protein-interaction interfaces. To rationalize the switch from a high-affinity *DC5con*-like to a high-cooperativity *DC5*-like binding configuration, we inspected the molecular environment of switch nucleotides in structural models. The ‘T3G8C9′ nucleotides are major features triggering the switch. T3 contacts Asn50 through van der Waals interactions (Figure 3F–H). G8 is directly contacted by Asn17 via hydrogen bond, G9′ (the base complementary to C9) is contacted by Gly18 through hydrogen bonds and finally T11 is contacted by the linker region residue Gly72 (30). Replacing G9′ by a *DC5*-like A9′ suggests a detrimental interaction with Gly18 as the N2 primary amine hydrogen donor is lost. Likewise, replacing G8 by C8 could de-stabilize the side chain Oδ of Asn-17 as it loses the H-bond donor amine at the position (Figure 3F, G and H). Replacing T11 to C (as in DC5) or G (as in N3) also leads to a less protein–DNA interactions in the region of the Pax6 linker, which was previously shown to be crucial for DNA binding (30). Hence, we surmise that on the high-affinity *pax* half-site, the ‘G8C9′ nucleotide in the *DC5con* sequence tightly tethers Pax6 to the minor groove of the DNA in a constrained configuration, in a manner that is independent of the influence of an adjacent Sox2-binding site. Hence, Sox2-Pax6 dimers are formed in an additive fashion. By contrast, a loss of this interaction by mutating G8C9 removes constraints and allows the Pax6 protein to adopt a conformation in favor of cooperative binding. The cooperative assembly on *DC5*-like sequences is likely facilitated by an allosteric, DNA-mediated mechanism. While structural models provide a rudimentary framework, the overall effect of these mutations in the degenerate *pax* half-sites on the binding topology and conformation of Pax6 is still ambiguous and will be made clear only when atomic resolution structures of Pax6 on *DC5*-like sequences become available (41).

A recent study on Sox2 and Pax6 cooperativity used plasmon resonance and gold nanoparticles tethered to the end of *DC5* and *DC5con* sequences. While Sox2 alone induced indistinguishable bends, it was reported that the *DC5* is more markedly deformed by Sox2–Pax6 dimers than the *DC5con* element (65). Apparently, Sox2–Pax6 binding induces structural changes to DNA in a sequence-dependent manner. Another study employed single molecule atomic force microscopy and reported cooperative binding as well as the requirement for Sox2-induced bending for effective complex formation (66). These studies are in accordance with a model where DNA deformations supportive of cooperative complex formation occur on the *DC5*-like sequence but not on *DC5con*-like sequences. However, a R75E mutation mapping to the C-terminal extension of the Sox2-HMG was demonstrated to abrogate Sox2–Pax6 dimerization on *DC5* (13). As this site interacts with Oct1 in the crystal structure of the Sox2/Oct1/Fgf4 ternary complex, the authors reasoned that this residue directly interacts with the Pax6-PRD as well (13). However, in our NMR experiments R75 could not be assigned due to pronounced line broadening. From our structural models, we observed that R75 is in proximity to the DNA backbone and is not far apart from the Pax6 PRD domain (Figure 5C). As our models on the *DC5* and *N3* enhancers suggest that the configuration of the ternary complexes needs to adapt to promote Sox–Pax cooperativity, it is possible that a glutamate at position 75 interferes with the adaptation. However, we cannot exclude that there might be some direct protein–protein interactions that do not lead to obvious chemical shifts in 2D-NMR. Yet, we surmise that a DNA-mediated mechanism is the main driver for the cooperative co-recruitment of Sox2–Pax6 to *DC5*-like sequences.

Genomic studies also point toward the versatile mechanism by which Pax6 engages chromatin. Analysis of the genome-wide binding of Pax6 by ChIPseq in human neuroectoderm revealed that less than 3% of the binding sites contain consensus *pax* motifs (Figure 9B). However, *sox* motifs were frequently found in proximity to Pax6-binding sites (Figure 9B). Moreover, missense mutations in either the PAI or RED subdomains differentially affect the genomic target selection of Pax6 (35). Apparently, some enhancers more strongly rely on PAI and others on RED for efficient genomic targeting. This suggests that Pax proteins employ different binding modes to recognize alternative sets of target sequences. Such differences could account for differential TF partner recruitment and profoundly affect the regulatory outcome of the binding event.

The striking observation that partner factors prefer sequences that are very different from their consensus motif creates another layer of complexity in understanding the principles underlying combinatorial regulation of transcription. A number of TF families are known to be able to accommodate diverse sets of binding sequences. Such binding modes pose specific challenges for motif scanning tools that rely on simple position weight matrices, which assume positional independence, or on consensus k-mer patterns from high-affinity sequence binders (67) or are too divergent to be captured by probabilistic models such as HMMs (68). Yet, given the critical roles of Sox and Pax TFs to direct cell fate choices and the frequent co-occurrence in the nuclei of diverse cell types, such unchartered non-consensus target sites are likely fundamentally important. Consistently, we detected eight novel target sites in human NSC enhancers where SOX2 and PAX6 cooperate. Therefore, an unbiased assessment of how TF partnerships influence DNA sequence preferences as well as an understanding of the structural basis for DNA-induced allostery is required to decode how developmental programs are genetically hard-wired.

## SUPPLEMENTARY DATA

Supplementary Data are available at NAR Online.

SUPPLEMENTARY DATA

## Accession Numbers

6PAX and 1GT0.
