# Supplementary material for: DNA-mediated cooperativity facilitates the co-selection of cryptic enhancer sequences by SOX2 and PAX6 transcription factors
Source: Nucleic Acids Res. 2015 Jan 10;43(3):1513–28. doi: 10.1093/nar/gku1390 (PMC4330359; doi:10.1093/nar/gku1390)
Supplement: SUPPLEMENTARY DATA [file supp_43_3_1513__index.html]

DNA-mediated cooperativity facilitates the co-selection of cryptic enhancer sequences by SOX2 and PAX6 transcription factors — DNA-mediated cooperativity facilitates the co-selection of cryptic enhancer sequences by SOX2 and PAX6 transcription factors — SUPPLEMENTARY DATA 

# DNA-mediated cooperativity facilitates the co-selection of cryptic enhancer sequences by SOX2 and PAX6 transcription factors

## SUPPLEMENTARY DATA

**Files in this Data Supplement:**

- SUPPLEMENTARY DATA
