# Supplementary material for: DNA-mediated cooperativity facilitates the co-selection of cryptic enhancer sequences by SOX2 and PAX6 transcription factors
Source: Nucleic Acids Res. 2015 Jan 10;43(3):1513–28. doi: 10.1093/nar/gku1390 (PMC4330359; doi:10.1093/nar/gku1390)
Supplement: SUPPLEMENTARY DATA [file supp_gku1390_nar-03404-x-2014-File011.pdf]

Supplementary Table 1. List of BP cloning primers used in this study. Forward and reverse primers are indicated as “primer name_f” and “primer name_r” respectively. Gene-specific sequences are underlined. Mutant sites are indicated in red color.

Primer name	Sequences
pax1_f	5'GGGGACAAGTTTGTACAAAAAAGCAGGCTTCGAAAACCTGTATTTTCAGGGCACGTACGGCGAAGTGAACCAACTTG3'
pax1_r	5'GGGGACCACTTTGTACAAGAAAGCTGGGTTTAGCTGCCAATCTTATTTCGCAGGATG3'
pax2_f	5'GGGGACAAGTTTGTACAAAAAAGCAGGCTTCGAAAACCTGTATTTTCAGGGCACGTACGGCGAAGTGAACCAACTTG3'
pax2_r	5'GGGGACCACTTTGTACAAGAAAGCTGGGTTTACTGCTGAACTTTGGTCCGGATGATCC
pax3_f	5'GGGGACAAGTTTGTACAAAAAAGCAGGCTTCGAAAACCTGTATTTTCAGGGCGACGTACGGCGAAGTGAACCAACTTG3'
pax3_r	5'GGGGACCACTTTGTACAAGAAAGCTGGGTTTATTTTCCAAATTTACTCCTCAGGATG3'
pax4_f	5'GGGGACAAGTTTGTACAAAAAAGCAGGCTTCGAAAACCTGTATTTTCAGGGCACGTACGGCGAAGTGAACCAACTTG3'
pax4_r	5'GGGGACCACTTTGTACAAGAAAGCTGGGTTTAGTCTTCCTGAAGTGCCCGAAGTAC3'
pax6_f	5'GGGGACAAGTTTGTACAAAAAAGCAGGCTTCGAAAACCTGTATTTTCAGGGCAGTCACAGCGGAGTGAATC3'
pax6_r	5'GGGGACCACTTTGTACAAGAAAGCTGGGTTTACTGTTGCTTTTCGCTAGC3'
pax8_f	5'GGGGACAAGTTTGTACAAAAAAGCAGGCTTCGAAAACCTGTATTTTCAGGGCGACGTACGGCGAAGTGAACCAACTTG3'
pax8_r	5'GGGGACCACTTTGTACAAGAAAGCTGGGTTTACTGCTGCACTTTGGTCCGGATGATTC3'
pax9_f	5'GGGGACAAGTTTGTACAAAAAAGCAGGCTTCGAAAACCTGTATTTTCAGGGCGACGTACGGCGAAGTGAACCAACTTG3'
pax9_r	5'GGGGACCACTTTGTACAAGAAAGCTGGGTTTAGTTGCCGATCTTGTTGCGCAGAATAC3'
Sox2_D123G_f	5'GACGCTCATGAAGAAGG**G**TAAGTACACGCTTCCC3'
Sox2 _D123G_r	5'GGGAAGCGTGTACTTA**C**CCTTCTTCATGAGCGTC3'
pax6_G36R_f	5'GAGCTAGCTCACAGC**C**GGGCCCGGCCGTGC3'
pax6_G36R_r	5'GCACGGCCGGGCCC**G**GCTGTGAGCTAGCTC3'
pax6_R44Q_f	5'GTGCGACATTTCCC**A**AATTCTGCAGGTATCC3'
pax6_R44Q_r	5'GGATACCTGCAGAA**T**TTGGGAAATGTCGCAC3'

Supplementary Table 2. List of DNA elements used in this study. The following DNA sequences labeled at the 5' end with 5-Carboxyfluorescein (FAM) or Cy5 were employed in the study. Sox half sites are colored blue and pax half sites are colored orange.

Name	Forward strand sequence
DC5	5'AAATATTCATTGTTGTTGCTCACCTACCATGGATCC3'
DC5con	5'AAATATTCATTGTTGATGTTCACGCATCATGGATCC3'
N3core	5'TCTTTTGTTTGGGATTACTGAGAGCTTAGCCTA3'
"-2"	5'TCTGAAATATTCATTGTTTGCTCACCTACCATGGATCC3'
"-1"	5'CTGAAATATTCATTGTTTTGCTCACCTACCATGGATCC3'
"+1"	5'GAAATATTCATTGTTGGTTGCTCACCTACCATGGATCC3'
"+2"	5'AAATATTCATTGTTGGGTTGCTCACCTACCATGGATCC3'
"+3"	5'AATATTCATTGTTGGGGTTGCTCACCTACCATGGATCC3'
"+4"	5'ATATTCATTGTTGGGGGTTGCTCACCTACCATGGATCC3'
"+5"	5'TATTCATTGTTGGGGGGTTGCTCACCTACCATGGATCC3'
Lama 1	5'ATCCAGGACAATAGAGACTGT3'
Pax6_xtal	5'AAGCATTTTCACGCATGAGTGCACAG3'
DC5_Pax6_site	5'GTTGTTGCTCACCTACCATGGACAAT3'
LE9	5'AAATATTAATTGATTTGAATGGGCAATGAGCGGAAA3'
G8	5'AAATATTCATTGTTGTTGCTCAC**G**TACCATGGATCC3'
C9	5'AAATATTCATTGTTGTTGCTCACC**C**ACCATGGATCC3'
T11	5'AAATATTCATTGTTGTTGCTCACCTA**T**CATGGATCC3'
T3	5'AAATATTCATTGTTGTTG**T**TCACCTACCATGGATCC3'
G8C9	5'AAATATTCATTGTTGTTGCTCAC**GC**ACCATGGATCC3'
T3G8C9	5'AAATATTCATTGTTGTTG**T**TCAC**GC**ACCATGGATCC3'
G8C9T11	5'AAATATTCATTGTTGTTGCTCAC**GC**A**T**CATGGATCC3'
T3C9T11	5'AAATATT**CATTGTT**GTTG**T**TCACC**C**A**T**CATGGATCC3'
T3T11	5'AAATATT**CATTGTT**GTTG**T**TCACCTA**T**CATGGATCC3'
ACACA	5'AGGTTTGTATTCATTCTTTTCAGCTTGCTTGGATTT3'
FGFR2	5'AAAGGATAATTGTGGTTTCTCAGTTACAAGCTCGAT3'
UPP2	5'CTTCTGTTTTTCTTTCAGCACGTGCATGAGGTCCAT3'
EFNA5	5'AGGTTTGTATTCATTCTTTTCAGCTTGCTTGGATTT3'
DC5con'-1	5'AAATATTCATTGTTGGTGCACTCATGCGTGAAATCC3'
DC5con'-2	5'AAATATTCATTGTTGTTGCACTCATGCGTGAAATCC3'

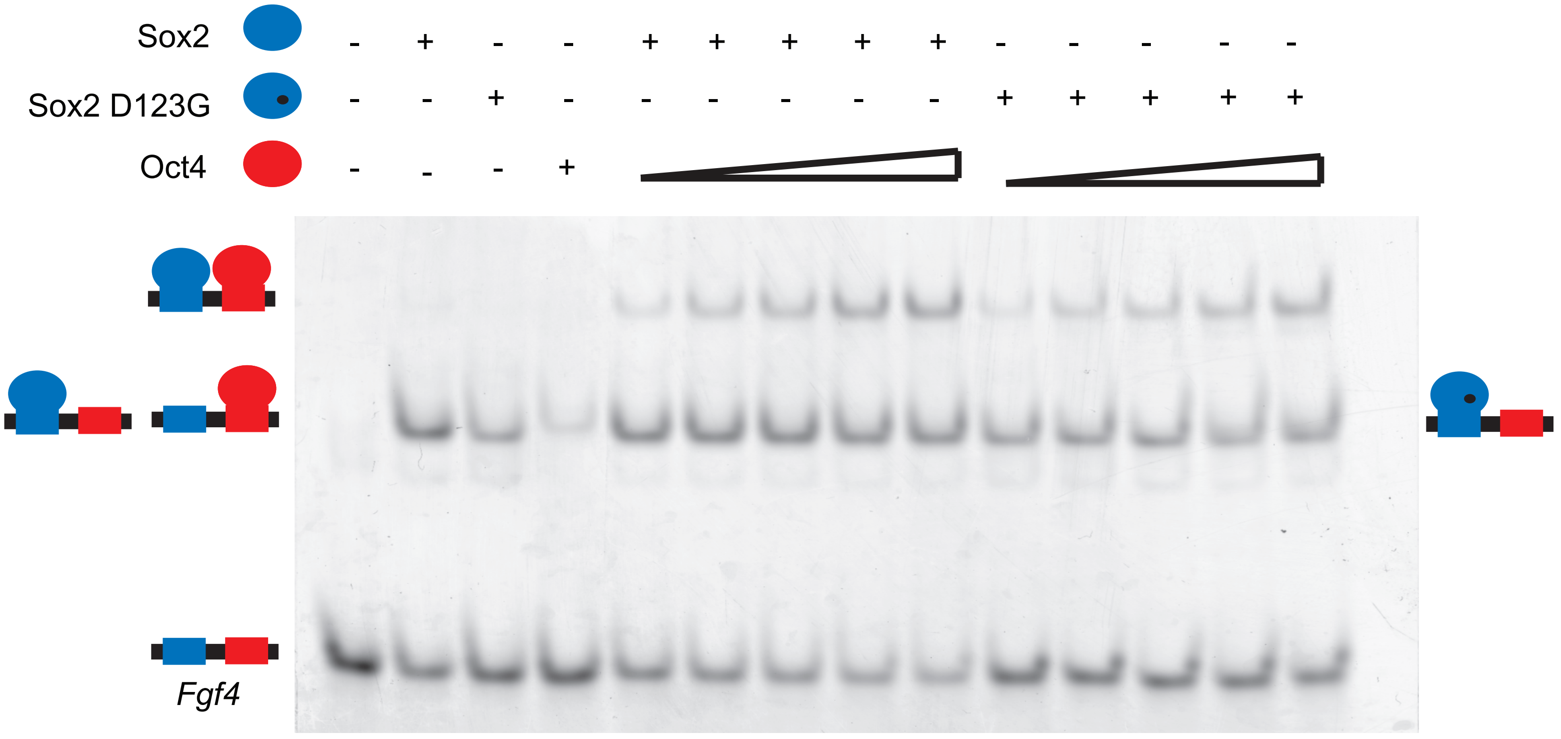

Supplementary Figure 1. Cooperativity of Sox2 and Oct4 is not altered due to point mutation D123G on Sox2.

EMSAs to compare the potential of Sox2 and Sox2 D123G to cooperate with Oct4 on Fgf4 DNA element. Cooperativity was not altered due to a point mutation D123G on Sox2. The cartoons on the left and right depicts the different binary and ternary complexes formed during EMSA.
